# Silencing the Transcriptional Repressor, ZCT1, Illustrates the Tight Regulation of Terpenoid Indole Alkaloid Biosynthesis in *Catharanthus roseus* Hairy Roots

**DOI:** 10.1371/journal.pone.0159712

**Published:** 2016-07-28

**Authors:** Noreen F. Rizvi, Jessica D. Weaver, Erin J. Cram, Carolyn W. T. Lee-Parsons

**Affiliations:** 1 Department of Chemical Engineering, Northeastern University, Boston, Massachusetts, 02115, United States of America; 2 Department of Biology, Northeastern University, Boston, Massachusetts, 02115, United States of America; 3 Department of Chemistry and Chemical Biology, Northeastern University, Boston, Massachusetts, 02115, United States of America; Southern Crop Protection and Food Research Centre, CANADA

## Abstract

The *Catharanthus roseus* plant is the source of many valuable terpenoid indole alkaloids (TIAs), including the anticancer compounds vinblastine and vincristine. Transcription factors (TFs) are promising metabolic engineering targets due to their ability to regulate multiple biosynthetic pathway genes. To increase TIA biosynthesis, we elicited the TIA transcriptional activators (ORCAs and other unidentified TFs) with the plant hormone, methyl jasmonate (MJ), while simultaneously silencing the expression of the transcriptional repressor ZCT1. To silence ZCT1, we developed transgenic hairy root cultures of *C*. *roseus* that expressed an estrogen-inducible *Zct1* hairpin for activating RNA interference. The presence of 17β-estradiol (5μM) effectively depleted *Zct1* in hairy root cultures elicited with MJ dosages that either optimize or inhibit TIA production (250 or 1000μM). However, silencing *Zct1* was not sufficient to increase TIA production or the expression of the TIA biosynthetic genes (*G10h*, *Tdc*, and *Str*), illustrating the tight regulation of TIA biosynthesis. The repression of the TIA biosynthetic genes at the inhibitory MJ dosage does not appear to be solely regulated by ZCT1. For instance, while *Zct1* and *Zct2* levels decreased through activating the *Zct1* hairpin, *Zct3* levels remained elevated. Since ZCT repressors have redundant yet distinct functions, silencing all three ZCTs may be necessary to relieve their repression of alkaloid biosynthesis.

## Introduction

The *Catharanthus roseus* plant is the source of many valuable terpenoid indole alkaloids (TIAs), including the anticancer compounds vinblastine and vincristine. Despite the low levels of these compounds in *C*. *roseus* (0.0002 wt%), these pharmaceuticals continue to be used for cancer treatments [1]. The structural complexity and complicated biosynthetic pathway of TIAs prohibit chemical synthesis or production in host systems at the commercial scale [2]. Instead, efforts to improve TIA supply focus on engineering the TIA biosynthetic pathway or its regulation in *C*. *roseus* cultures. In particular, *C*. *roseus* hairy root cultures are model systems for studying the production of TIAs due to their genetic and biochemical stability, and fast growth in hormone-free media.

**Fig 1:**
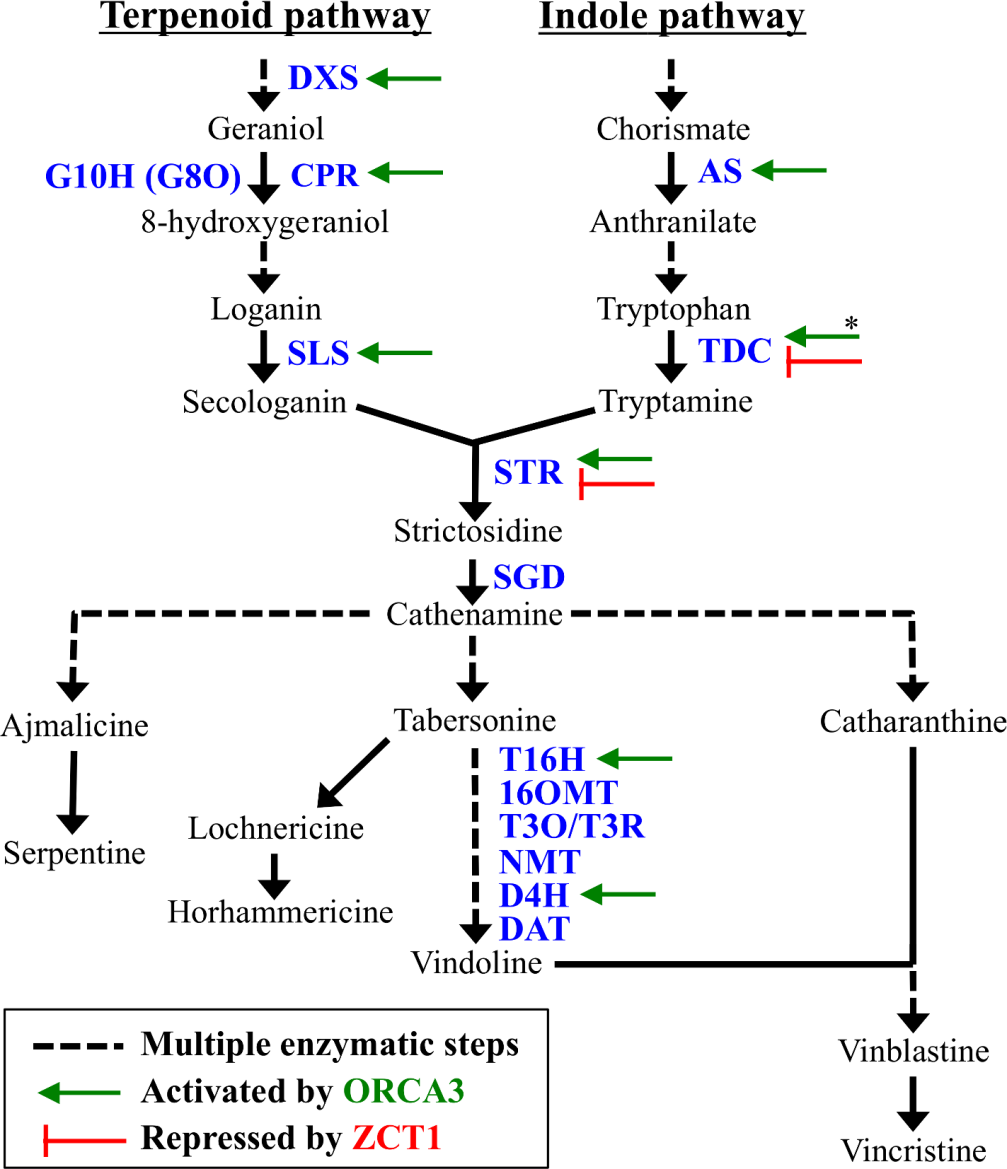
Terpenoid indole alkaloid (TIA) biosynthesis in *C*. *roseus*. Solid arrows indicate single step, whereas dashed arrows represent multi-step enzymatic conversions. Enzymes activated by ORCA3 and/or repressed by ZCT1 (based on either promoter binding, transactivation, or overexpression studies) are indicated by a green arrow or red stop, respectively [13–16]. *Binding/transactivation studies show ORCA2 and ORCA3 are activators of TDC, but only activate TDC in cell suspensions, not hairy roots. DXS = 1-deoxy-D-xylulose-synthase; CPR = cytochrome P450 reductase; G10H (G8O) = geraniol-10-hydroxylase (or geraniol 8-oxidase); SLS = secologanin synthase; AS = anthranilate synthase, α/β subunits; TDC = tryptophan decarboxylase; STR = strictosidine synthase; SGD = strictosidine β-D-glucosidase; T16H = tabersonine 16-hydroxylase; 16OMT = 16*-O*-methyltransferase; T3O = tabersonine 3-oxygenase; T3R = tabersonine 3-reductase; NMT = *N*-methyltransferase; D4H = desacetoxyvindoline 4-hydroxylase; DAT = deacetylvindoline 4-O-acetyltransferase.

TIAs are the condensation products of two precursor pathways. Tryptamine from the indole pathway and secologanin from the terpenoid pathway condense to form strictosidine, the backbone of TIAs (Fig 1). Initial precursor feeding analysis suggested that tryptamine or secologanin likely limited TIA production [3–8]. Therefore, early genetic engineering strategies focused on overexpressing biosynthetic enzymes in those precursor branches, particularly the first committed steps of each branch (S1 Table). Genetic manipulations of the indole pathway (such as *Asα*, *Tdc*, *Asα + Tdc*, *Asα + Asβ*, or *Asα + Asβ + Tdc;* see Fig 1) increased the TIA precursors, tryptophan and/or tryptamine, but did not lead to large increases in downstream TIAs [9–11]. Similarly, overexpression of key enzymes in the terpenoid pathway (such as *Dxs* and *G10h*, also known as *G8o*) did not considerably increase TIA levels [12].

Transcription factors (TFs) are promising metabolic engineering targets due to their ability to regulate multiple biosynthetic pathway genes [17]. Therefore, recent efforts to increase TIA levels in *C*. *roseus* have focused on engineering the transcriptional regulation of TIAs through TFs [13,18]. The transcription factors regulating TIA biosynthesis include the activators ORCA2, ORCA3, BIS1, BPF1, MYC1, MYC2, and WRKY1 [14,19–25] and the repressors JAZ, ZCT1, ZCT2, ZCT3, GBF1, and GBF2 [15,26–28]. Jasmonate (JA, or methyl jasmonate, MJ), a phytohormone produced in a defense response, activates the signaling cascade, which induces transcription factors that regulate TIA biosynthetic enzymes. Our research focuses on the ORCA and ZCT transcription factors since they are MJ-responsive, whereas other downstream TFs, such as WRKY1 and GBF, are not [16].

The ORCA (octadecanoid-responsive *Catharanthus* AP2/ERF domain) transcription factors, including ORCA2 and ORCA3, are well-known transcriptional activators of several biosynthetic genes in *C*. *roseus* (Fig 1). The overexpression of *Orca3* in cell cultures increased a subset of TIA biosynthetic genes (i.e. *Dxs*, *Cpr*, *Asα*, *Tdc*, *Str*, and *D4h*) [14]. Since *G10h* was not expressed, TIA production did not increase except upon addition of loganin. When *Orca3* was overexpressed in *C*. *roseus* hairy roots, TIA levels did not significantly increase (even with loganin addition) despite increased expression of several biosynthetic genes (i.e. *Dxs*, *Asα*, *Sls*, and *Str*) [13]. Similarly, overexpression of *Orca2* in hairy roots increased the levels of certain metabolites (i.e. tryptamine, 16-hydroxytabersonine, and 19-hydroxytabersonine) while other metabolites decreased (i.e. tabersonine, strictosidine, and horhammericine) [29] even with significant increases in the expression of specific biosynthetic genes (i.e. *Str*, *T16h*, and *D4h*). Despite the increases in biosynthetic gene expression upon *Orca* overexpression, elicitation with JA alone produced a larger increase on TIA levels [13], since several biosynthetic genes are not regulated by ORCA [14]. These results suggest that other transcriptional activators are likely involved in the JA-induced expression of these TIA genes. Recently, an additional JA-inducible transcriptional activator, bHLH iridoid synthesis 1 (BIS1), was discovered and shown to regulate the expression of steps in the terpenoid pathway between geranyl diphosphate and loganic acid [25]. The overexpression of BIS1 increased TIA levels, including strictosidine, tabersonine, ajmalicine, and serpentine, specifically in cell suspensions.

Three members of the Cys_2_/His_2_-type (transcription factor IIIA-type) zinc finger protein family, ZCT1, ZCT2, and ZCT3, are transcriptional repressors that inhibit the expression of *Tdc* and *Str* [15]. ZCT proteins counteracted the transcriptional activation of *Tdc* and *Str* by ORCAs [15]. In *C*. *roseus* hairy roots, optimum MJ dosages favored high transcript levels of *Orca* to *Zct* while inhibitory MJ dosages repressed TIA levels and induced high transcript levels of *Zct* to *Orca* [16]. While MJ induced all three *Zcts*, *Zct1* responded most strongly to increased MJ. Furthermore, overexpression of *Orca2* [29] or *Orca3* [13,30] activated the expression of all three *Zcts*, especially *Zct1* and *Zct2*. Therefore, our strategy to enhance TIA biosynthesis involves MJ to induce TIA genes through ORCAs and other unidentified TFs while simultaneously silencing ZCT1 to counter its activation by MJ and ORCA.

In this study, we explored the effect of silencing *Zct1* in *C*. *roseus* hairy roots elicited with MJ. We employed a previously characterized estradiol-inducible system [31] to successfully control timing of *Zct1* silencing, and analyzed the effect of *Zct1* silencing on TIA production. To further understand the regulation of TIAs in *C*. *roseus*, we monitored the expression of biosynthetic genes and other transcription factors. *Zct1* silencing did not affect TIA biosynthesis or the expression of biosynthetic genes and transcription factors, illustrating the tight regulation of TIA biosynthesis.

## Materials and Methods

### Preparation of pER8-Zct1hp and pER8-GFPhp plasmids and electroporation into *Agrobacterium rhizogenes*

pER8-GFP and pSK-Int were obtained from Dr. Nam-Hai Chua (The Rockefeller University). The pSK-Int vector is an intermediate cloning plasmid for generating hairpin RNAi constructs. It contains the third intron from the *Arabidopsis* actin-11 gene with a multiple cloning site (MCS) on each side of the intron. Two 163bp fragments of *Zct1* (Genbank accession AJ632082) were amplified from *C*. *roseus* cDNA using primers containing restriction sites and cloned into each of the MCSs to generate a hairpin in the pSK-Int vector (S2 Table). The *Zct1* hairpin (Zct1hp) was subsequently removed from pSK-Int and cloned into the pER8 backbone using restriction cloning (XhoI-SpeI) (S1 Fig). A GFP-hairpin construct (GFPhp) in pUC57(kan) was synthesized by GENEWIZ containing the same actin-11 intron from pSK-Int. A SpeI site and XhoI site flanks the hairpin construct on opposite sides (S2 Table). The GFP-hairpin construct was moved from pUC57(kan) to pER8 using restriction cloning.

DH5α *Escherichia coli* competent cells were used for cloning (Z-competent *E*. *coli* Transformation Kit, G-Biosciences). *E*. *coli* was grown in LB media (10 g/L tryptone, 5 g/L yeast extract, 10 g/L NaCl with 15 g/L agar for plates, pH = 7.0) at 37°C overnight at 250 rpm when in liquid culture. The pER8-Zct1hp and pER8-GFPhp constructs were electroporated into *Agrobacterium rhizogenes* R1000 (ATCC 43056) as previously described, except LB media was used instead of YM [31]. R1000 was grown at 26°C for 2–3 days at 250 rpm when in liquid culture. See S2 Table for antibiotic resistance conferred by each vector and concentrations used.

### Transformation of *C*. *roseus* seedlings with *A*. *rhizogenes*

*C*. *roseus* seedlings were germinated from seeds (Vinca Little Bright Eye, Neseed, Hartford, CT), grown aseptically, and transformed with *A*. *rhizogenes* R1000 containing pER8-Zct1hp, as previously described [31]. Of the 428 individual roots tested, 144 survived two rounds of selection on hygromycin for an overall transformation efficiency of 33.6% (Table 1). Similarly, 255 GFPhp roots were generated, of which 71 survived two rounds of selection, resulting in a transformation efficiency of 27.8%. These efficiencies are comparable to previously reported efficiencies using this optimized *Agrobacterium*-mediated transformation method and estrogen-inducible construct [31].

**Table 1:** Overall transformation efficiency of transgenic Zct1hp and GFPhp *C*. *roseus* hairy roots after two rounds of hygromycin selection.

Line	Total # roots tested	# roots surviving first selection (5mg/L hygromycin)	# roots surviving second selection (15mg/L hygromycin)	Overall Efficiency
Zct1hp	428	215	144	33.6%
GFPhp	255	117	71	27.8%

Of these 144 transgenic Zct1hp lines, 26 well-growing lines were originally adapted to liquid culture, of which 8 lines survived long-term subculture in liquid (over 16 subcultures at the time of publication). Of the 71 transgenic GFPhp lines, 36 were originally adapted to liquid culture, of which 10 have survived long-term subculture in liquid (6–7 subcultures at the time of publication) and were subcultured every 28-days and maintained as previously reported [16]. Hairy root pieces (3 cm) were inoculated into sterile 125-mL flasks containing 25 mL liquid half-strength Gamborg’s media (30 g/L sucrose, 1.55 g/L Gamborg’s B-5 salts, 1 mL/L 1000X Gamborg’s vitamins, pH = 5.7). The adaptation of cultures to liquid is reportedly a difficult and limiting step in generating transgenic *C*. *roseus* hairy roots [32].

### Genomic DNA extraction and PCR

From the transgenic lines originally transferred to liquid culture, integration of the transgenes was verified in 10 Zct1hp lines and 9 GFPhp lines (remaining lines not checked; S2 Fig). Genomic DNA was extracted from wild-type (WT; *C*. *roseus* hairy root cultures generated by transformation with *A*. *rhizogenes* strain 15834 without any additional plasmids) and transgenic *C*. *roseus* roots using the CTAB method as previously described [31]. PCR was used to amplify specific genes from the gDNA using primers designed for *Rps9* (the housekeeping gene), *LexA*, *hygR*, *virD*, and *rolC* (S3 Table). The thermocycler protocol consisted of a heating step at 95°C for 10 min, then 30 cycles of 95°C for 30 s, 60°C for 45 s, and 72°C for 1 min. After 30 cycles, the extension step at 72°C was repeated for another 10 min. PCR products were run on a 2% agarose gel and viewed under a UV transilluminator to verify product sizes. The products were extracted from the gel using Zymoclean Gel DNA Recovery Kit (Zymo Research Corporation) and sequenced to confirm the correct product (GENEWIZ, Boston).

All transgenic Zct1hp and GFPhp lines have *Rps9* (*C*. *roseus* housekeeping gene), *LexA* (promoter for the chimeric XVE transcription factor, part of the estrogen-inducible construct), *hygR* (hygromycin-resistance gene used for selection), and *rolC* (hairy root control, *rol* genes are essential for hairy roots formation; S2 Fig). None of the lines (except faint band in Zct1hp-12) have *virD2* (*Agrobacterium* specific virulence gene), which indicates the successful elimination of *Agrobacterium* and confirms the transgenes are not due to any contaminating *Agrobacterium*.

### Induction of transgenic hairy roots with estradiol and/or MJ

To express the silencing hairpin (Zct1hp or GFPhp), transgenic *C*. *roseus* hairy roots were induced with 5μM 17β-estradiol (Fisher Scientific) on day 26 (late-exponential growth phase). Previously, induction with 5μM 17β-estradiol showed strong and tightly regulated expression of GFP in control transgenic *C*. *roseus* hairy roots [31]. Stock solutions (5mM) of 17β-estradiol were prepared in dimethyl sulfoxide (DMSO, Sigma Aldrich), and 50μL of stock solution was added to 50mL of culture media to achieve a final concentration of 5μM 17β-estradiol. Uninduced cultured were treated with 50μL of DMSO.

Cultures were elicited with MJ (≥ 95%, Sigma Aldrich) 24 h after induction with 17β-estradiol. Stock solutions were prepared in ethanol (200 proof, ACS/USP grade, Pharmco-AAPER) and added to 50mL of culture media to achieve final concentrations of 250μM or 1000μM MJ. Cultures were harvested 8, 24, and 48 h after MJ addition for mRNA analysis or after 3, 5, and 7 days for TIA metabolite analysis. *C*. *roseus* hairy roots were blotted and flash-frozen in LN_2_.

### Extraction of TIA metabolites from *C*. *roseus* hairy root cultures

TIAs were extracted from transgenic *C*. *roseus* hairy root cultures as previously described [16]. In short, frozen hairy root cultures were lyophilized using a Flexi-Dry MP Freeze-Dryer (Kinetics Thermal Systems). Root cultures were pulverized using a mortar and pestle and ~50mg of dried root powder was extracted using 5mL of methanol (HPLC grade) twice. The extracts were pooled and concentrated overnight (Savant SpeedVac Plus Concentrator, Thermoquest). The dried alkaloid-containing extracts were then re-dissolved in 1mL of methanol (HPLC grade) and filter-sterilized using non-sterile syringe filters (Millipore Millex Nonsterile Syringe Filters) into HPLC vials (Waters Corp.).

### TIA metabolite analysis by HPLC

TIA levels in the *C*. *roseus* hairy root extracts were analyzed through HPLC (Waters 2695 Separations Module, Waters 996 Photodiode Array Detector, Empower 2 Software) and separated using a reversed-phase C18 column (Luna, 150 x 4.60mm ID column, 5μm particle size, Phenomenex).

The HPLC mobile phases for TIA separation were: 99.9% water with 0.1% (v/v) formic acid as the aqueous phase, and 99.9% acetonitrile with 0.1% (v/v) formic acid as the organic phase. The protocol consisted of the following steps: 1) 90% aqueous and 10% organic as the initial condition, 2) gradient to 70% aqueous and 30% organic over 20 minutes, 3) gradient to 100% organic over 8 minutes, 4) gradient to 90% aqueous and 10% organic over 10 minutes, and 5) isocratically at 90% aqueous and 10% organic for 20 minutes to equilibrate the column for the next injection. All the flow rates were maintained at 1.0 mL/min.

Strictosidine (monitored at 274 nm; a gift from Dr. Sarah O’ Connor, John Innes Centre, Norwich, UK), ajmalicine (254 nm; TCI America, Portland, OR), serpentine (254 nm; Sigma-Aldrich), tabersonine-like peak 5 (329 nm), tabersonine-like peak 6 (329 nm), and tabersonine (329 nm; a gift from Prof. Martin E. Kuehne, University of Vermont, Burlington, VT) were monitored at the respective wavelengths and quantified with calibration curves using pure standards or Beer’s Law correlations (for strictosidine). The UV absorbance spectrum of Peak 5 and 6 were similar to that of tabersonine (S3 Fig). MS analysis was previously performed to verify the known compounds [8] and are in progress for identifying the compounds associated with peak 5 and 6.

### RNA extraction and gene expression analysis by qPCR

Transcript levels of transcription factor and TIA biosynthetic genes were monitored in the transgenic *C*. *roseus* hairy root cultures by qPCR. mRNA was extracted from frozen hairy root cultures using the RNAzol®RT (Molecular Research Center) method and quantified using a NanoDrop (ND-1000 Spectrophotometer; ThermoScientific). Extracted RNA was treated with DNase to remove genomic DNA, and cDNA was synthesized from the mRNA (1–5 μg; Deoxyribonuclease I Amplification Grade, SuperScript First-Strand Synthesis System for RT-PCR, Invitrogen).

Transcription factor genes (*Orca2*, *Orca3*, *Zct1*, *Zct2*, and *Zct3*) and TIA biosynthetic genes (*G10h*, *Tdc*, and *Str*) were monitored using the primers listed in S4 Table. qPCR was performed using the RT^2^ Real-Time^TM^ SYBR Green/ROX PCR master mix (SABiosciences) and QuantStudio 6 (Applied Biosystems) using the thermocycler protocol previously described [16]. The amplification efficiency for each gene was calculated using Ct values over a range of cDNA dilutions and was ~100% for each gene monitored. Fold changes were calculated using the ∆∆Ct method.

## Results and Discussion

### Transgenic hairy root cultures exhibited low levels of *Zct1* upon induction of the *Zct1* silencing construct

The estrogen-inducible XVE system is made up of an artificial XVE transcription factor containing the DNA-binding domain of the bacterial repressor Le**x**A, the Herpes **V**P16 activation domain, and the carboxyl region of the human **e**strogen receptor [33]. The XVE transcription factor is only activated in the presence of 17β-estradiol, which then promotes binding to the *LexA* operator sequence to induce transgene expression. The estrogen-inducible XVE system has been shown to be a tightly regulated and highly inducible system in *C*. *roseus* hairy roots [31]. Therefore, we established transgenic *C*. *roseus* hairy root cultures with estrogen-inducible expression of the *Zct1* hairpin to induce RNA interference (S1 Fig, referred to as Zct1hp in this text). Additionally, we established *C*. *roseus* hairy roots with estrogen-inducible silencing of green fluorescent protein (GFP) as a control for the effects of RNAi (referred to as GFPhp in this text).

Of the 428 Zct1hp hairy roots generated, 144 survived two rounds of selection on hygromycin and 26 of these were transferred to liquid media. Of the 255 GFPhp roots generated, 71 survived two rounds of hygromycin selection and 36 of these were transferred to liquid media (Table 1). We verified the genomic integration of transgenes in 10 Zct1hp and 9 GFPhp lines, which grew well in liquid media (S2 Fig).

**Fig 2:**
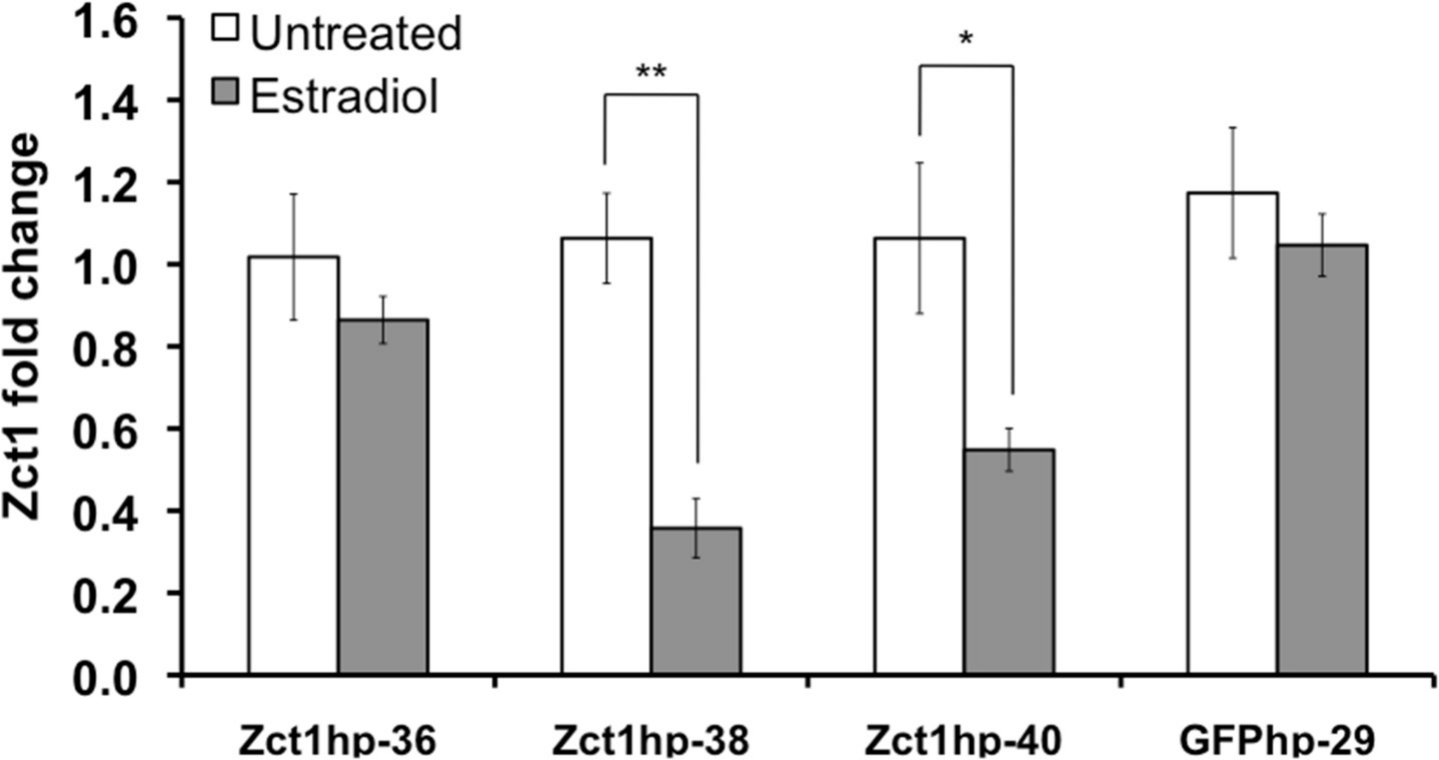
*Zct1* expression in Zct1hp-36, Zct1hp-38, Zct1hp-40, and GFPhp-29 transgenic lines with 5μM 17β-estradiol treatment for 24 h. Fold change is calculated with respect to each line’s untreated control. Error bars represent standard deviations of biological triplicates. Statistical significance calculated used Student’s t-test; * *p* < 0.05, ** *p* < 0.005.

We assessed the levels of *Zct1* silencing under estradiol induction in transgenic *C*. *roseus* hairy root cultures through qPCR. 17β-estradiol (5μM) was added to three individual Zct1hp lines (Zct1hp-36, Zct1hp-38, Zct1hp-40) for 24 h since estrogen-inducible gene expression was high at this concentration and timepoint [31]. *Zct1* silencing varied between the three estradiol-induced Zct1hp lines, with the *Zct1* levels ranging from 0.35- to 0.9-fold of their untreated controls (Fig 2). For example, Zct1hp-38 showed strong silencing (0.35-fold), but Zct1hp-36 was not significantly silenced (~0.9-fold) upon induction with 17β-estradiol. The variability in *Zct1* silencing is likely attributed to gene copy number and/or positional effects associated with the random T-DNA integration of *Agrobacterium*-mediated transformations. In our previous publication, we also observed variable GFP expression using the same estrogen-inducible XVE system to express GFP [31]. Neither 17β-estradiol nor the artificial transcription factor, XVE, altered the expression of *Zct1*. Furthermore, *Zct1* expression levels were not affected by 17β-estradiol in the GFPhp controls, indicating that the induction of RNAi also did not induce *Zct1* expression (Fig 2).

Based on these results, we selected two transgenic lines, Zct1hp-38 and Zct1hp-40, showing the strongest inducible silencing of *Zct1* for further studies to determine the effect of *Zct1* silencing on the regulation of TIA biosynthesis. The results of Zct1hp-38, which showed the strongest silencing, are presented in the following sections. The results for Zct1hp-40 are consistent with that of Zct1hp-38 and demonstrate the reproducibility of the trends observed with *Zct1* silencing.

### *Zct1* induction by MJ was abolished in *Zct1* silenced lines

MJ induces TIA production through the action of several transcriptional activators (MYC2, ORCA2, ORCA3, and BIS1) [14,19,20,23,25]. But MJ also induces the expression of the ZCT transcriptional repressors [15,16]. The MJ dosage of 250μM promoted a high *Orca* to low *Zct* level and optimized TIA production in hairy root cultures, while the MJ dosage of 1000μM resulted in a high *Zct* to low *Orca* level and inhibited TIA production [16]. Therefore, we examined whether *Zct1* was effectively silenced with estradiol at both optimum and inhibitory MJ dosages. Based on our previous publications, 17β-estradiol (5μM) was first added to Zct1hp lines for 24 h, followed by the addition of either 250μM or 1000μM MJ for 8, 24, and 48 h [16,31]. *Zct1* expression was monitored through qPCR.

**Fig 3:**
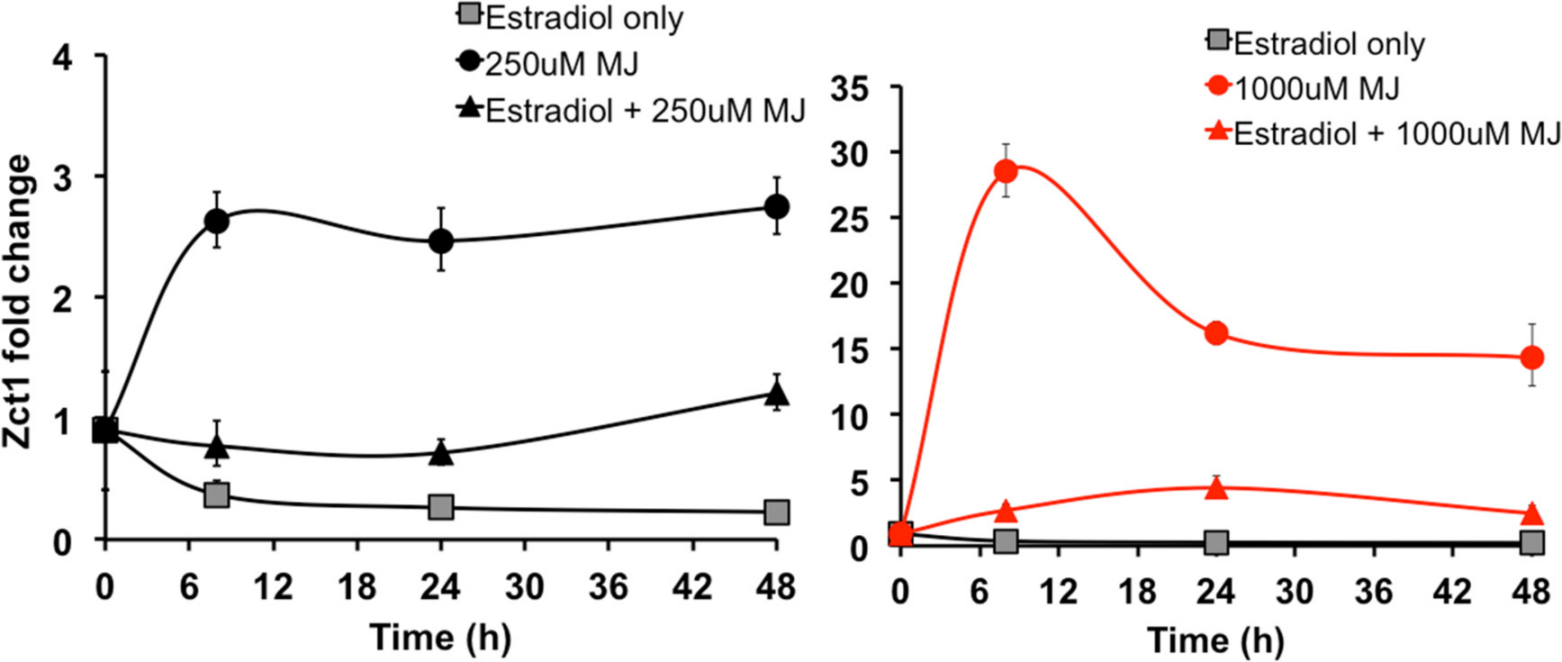
*Zct1* expression in Zct1hp-38 over 48 h. 17β-estradiol (5μM) was added for 24 h, then 250μM or 1000μM MJ was added for the time specified (8, 24, and 48 h). Error bars represent standard deviations of qPCR triplicates.

Induction with 17β-estradiol alone successfully decreased expression of *Zct1* in Zct1hp-38 line, and this decrease was sustained over 48 h (Fig 3). *Zct1* levels increased by 2.8-fold upon elicitation with 250μM MJ and by 28-fold upon elicitation with 1000μM MJ in Zct1hp-38, similar to that previously reported [16]. However, upon treatment with 17β-estradiol and 250μM MJ, the increase of *Zct1* attributed to MJ was completely abolished (Fig 3). Similarly, treatment with 17β-estradiol and 1000μM MJ reduced the induction of *Zct1* to less than 5-fold. This indicates that *Zct1* silencing can effectively knock-down the induction of *Zct1* by MJ. This effect was also seen in the Zct1hp-40 line (S4 Fig). Importantly, in the GFPhp-29 control line, *Zct1* expression remained unchanged upon induction of RNAi with 17β-estradiol (S4 Fig).

### MJ-elicited TIA production did not further increase upon *Zct1* silencing

As shown in S2 Fig, we verified the genomic integration of transgenes in 10 Zct1hp and 9 GFPhp lines. We randomly selected and screened the TIA production of 6 out of the 10 Zct1hp lines to determine if *Zct1* silencing could enhance the production of TIAs in MJ-elicited cultures. Zct1hp lines were treated with 5μM 17β-estradiol for 24 h, followed by 250μM MJ for 5 d when alkaloid production reached maximum [16]. The TIAs were separated by HPLC and quantified by UV absorbance (S3 Fig).

**Fig 4:**
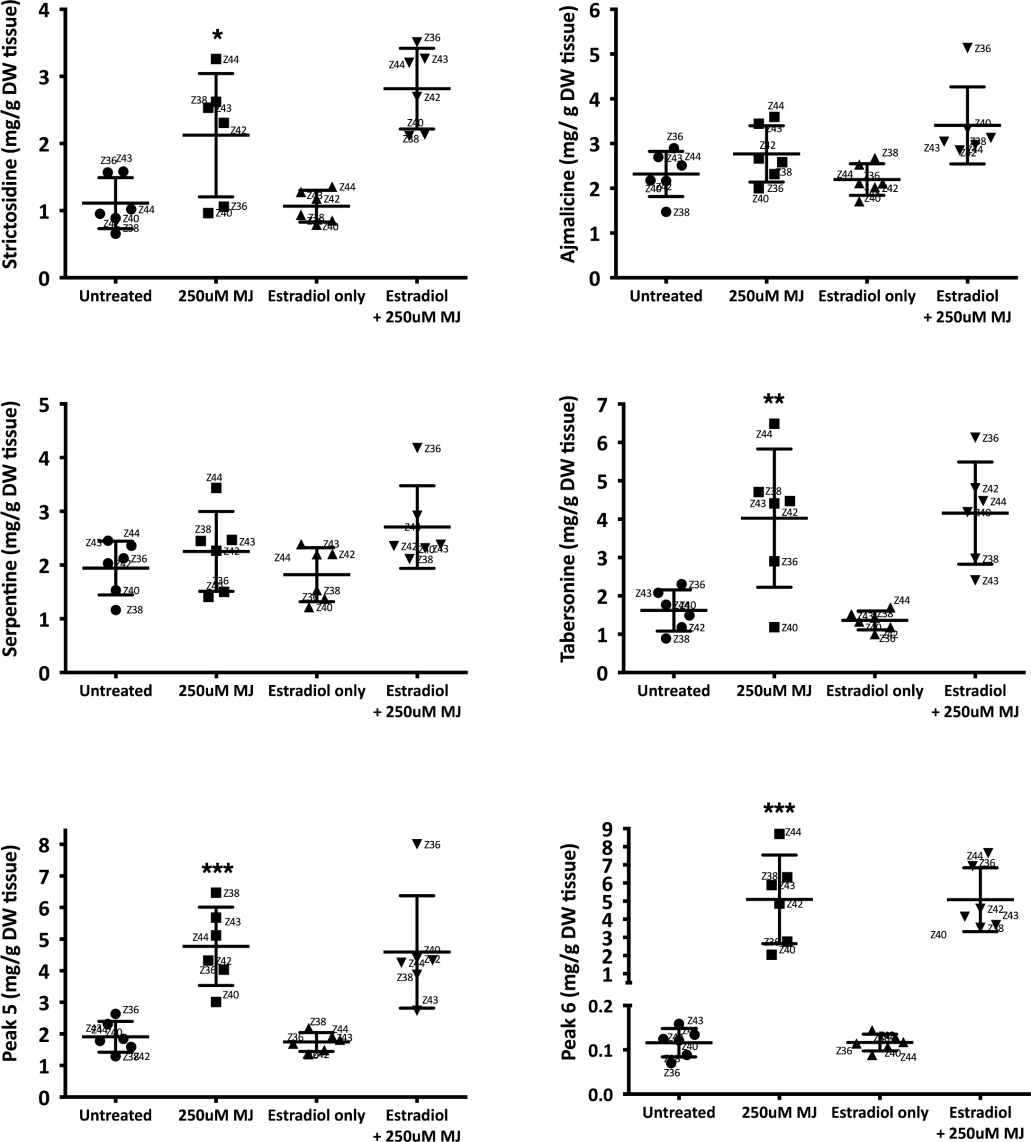
Average TIA metabolite levels across six Zct1hp lines (Zct1hp-36, -38, -40, -42, -43, -44). 17β-estradiol (5μM) was added for 24 h, then 250μM MJ was added for 5 days. The TIAs were separated by HPLC and quantified by UV absorbance. Each data point represents the TIA level of one biological replicate; variability between biological replicates for a specific line is shown in Fig 5. Bars represent the mean and standard deviations of the six lines. Statistical significance calculated used Student’s t-test; * *p* < 0.05, ** *p* < 0.01, *** *p* < 0.0005.

The distribution of TIA levels and the average across six Zct1hp lines are shown (Fig 4). None of the metabolites are affected by treatment with 17β-estradiol alone, suggesting the important role of MJ in eliciting transcriptional activators and TIA production. With 250μM MJ, levels of several metabolites increased as expected, including strictosidine (*p* = 0.03), tabersonine (*p* = 0.01), and representative tabersonine-like compounds (peak 5 and 6, *p* = 0.0005), but no further increases occurred with both 17β-estradiol and 25003B0043M MJ (Fig 4). Overall, *Zct1* silencing did not increase TIA production above the addition of MJ alone.

In this study, we see that the overall flux is largely responsive to MJ treatment, but the silencing of *Zct1* does not further increase the metabolic flux. The dominating effect of MJ over genetic engineering manipulations has been seen before [13]. This work emphasizes the need to further elucidate the complex regulatory network by which MJ induces TIA gene expression and metabolite production, as recently illustrated [34].

### TIA production inhibited upon 1000μM MJ induction, despite *Zct1* silencing

TIA metabolite levels did not increase further upon *Zct1* silencing combined with 250μM MJ. However, *Zct1* levels were low at 250μM MJ relative to 1000μM MJ (2.8-fold compared to 28-fold, respectively; Fig 3). To investigate the effect of *Zct1* silencing when *Zct* levels are high, we analyzed TIA metabolite levels in Zct1hp roots treated with 17β-estradiol and 1000μM MJ. We showed that the induction of *Zct1* silencing at 1000μM MJ reduced the *Zct1* levels in the Zct1hp cultures (Fig 3) and would expect TIA levels to be affected. To test this idea, we treated Zct1hp-38 with 5μM 17β-estradiol for 24 h, followed by 250μM MJ or 1000μM MJ addition for 3 and 5 d.

**Fig 5:**
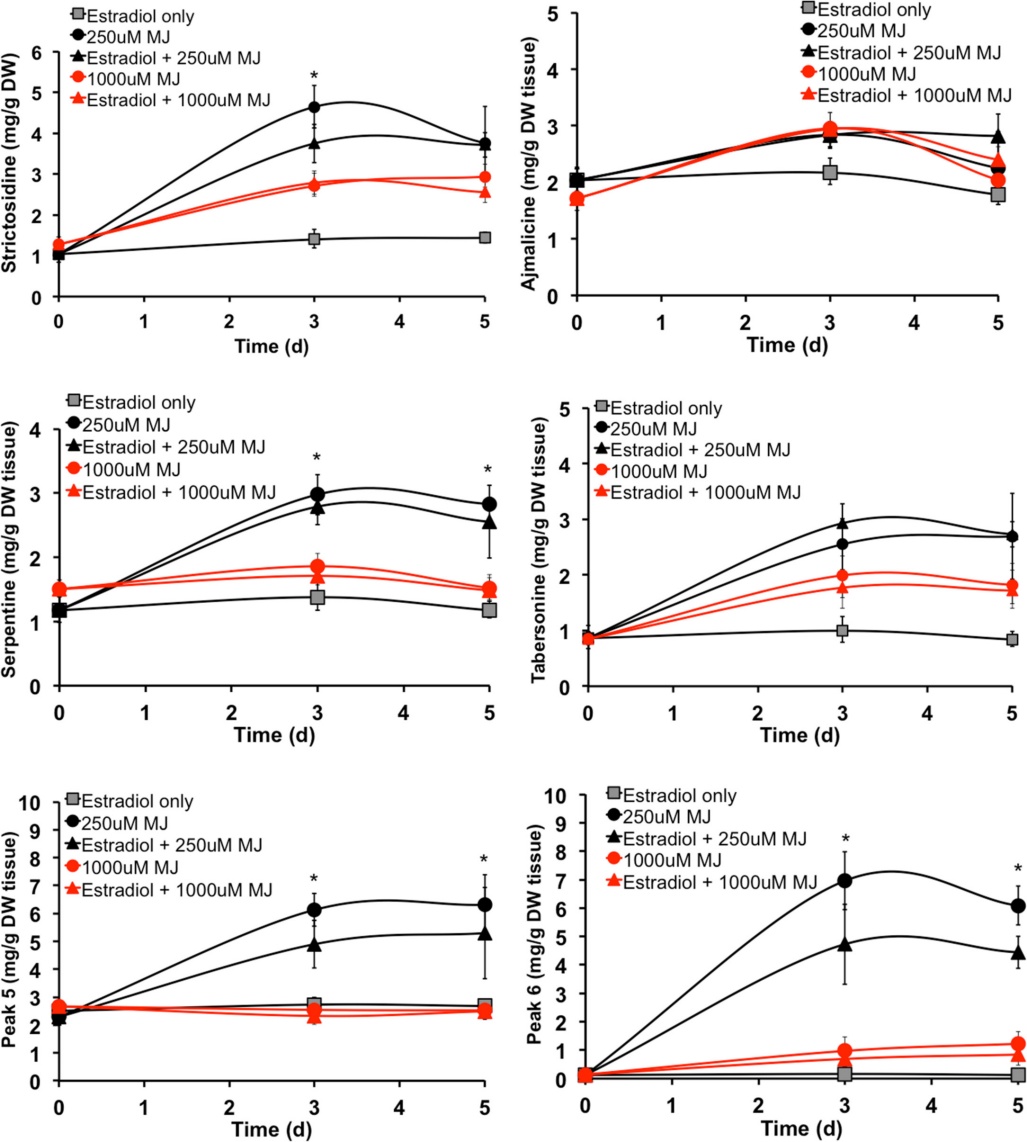
TIA metabolite levels in Zct1hp-38. 17β-estradiol (5μM) was added for 24 h, then 250μM or 1000μM MJ was added for the time specified (3 and 5 d). The TIAs were separated by HPLC and quantified by UV absorbance. Error bars represent standard deviations between two biological replicates.

Upon induction with 1000μM MJ (Fig 5), levels of strictosidine, serpentine, and tabersonine-like compounds (peak 5 and 6) were all significantly lower than at 250μM MJ induction; this inhibition at 1000μM MJ has been previously reported [16]. Surprisingly, levels of these TIAs still did not increase with 17β-estradiol and 1000μM MJ treatment, even though *Zct1* levels were low and similar to the *Zct1* levels at 250μM MJ treatment (Fig 3). TIA production also did not increase in the Zct1hp-40 line with the combination of 17β-estradiol and 1000μM MJ compared to 1000μM MJ alone (S5 Fig). This suggests that the mechanism by which TIA production is repressed at 1000μM MJ is not solely regulated by ZCT1.

### MJ-elicited TIA gene expression also did not further increase upon *Zct1* silencing

To understand why TIA levels did not further increase with *Zct1* silencing, we analyzed the expression of several TIA biosynthetic genes: G10H and TDC catalyze the first committed steps of the terpenoid and indole pathways, respectively, and STR catalyzes the condensation reaction leading to the common TIA backbone, strictosidine. ZCTs are shown to bind and repress the expression of *Tdc* and *Str* [15] using *in vitro* binding and transient expression assays. Here, we monitor the role of ZCT1 in repressing *G10h*, *Tdc*, and *Str* in stably transformed roots expressing the inducible *Zct1* hairpin. 17β-estradiol (5μM) was added to Zct1hp-38 for 24 h, followed by 250μM or 1000μM MJ for 8, 24, and 48 h [16]. The expression of *G10h*, *Tdc*, and *Str* was monitored by qPCR.

**Fig 6:**
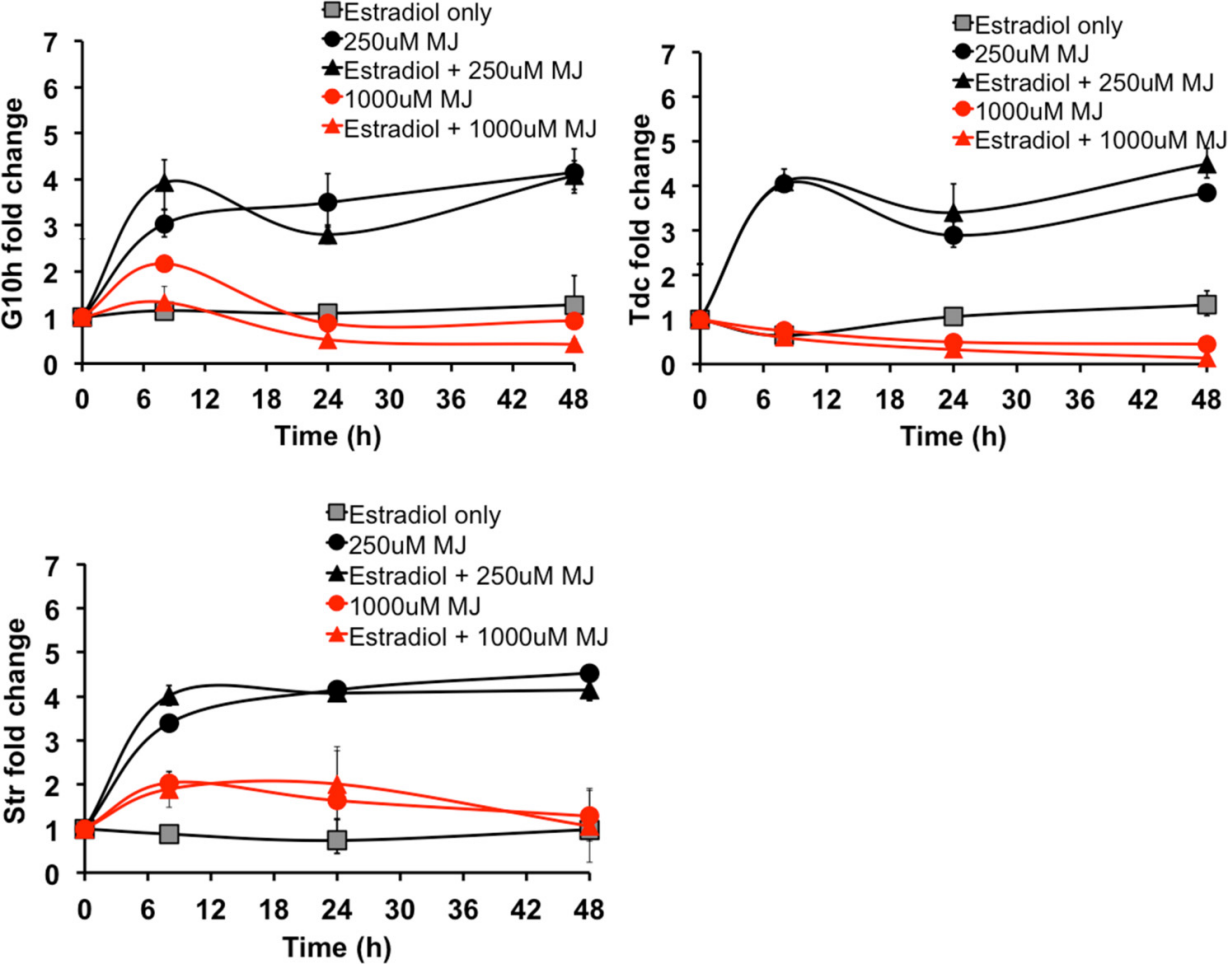
*G10h*, *Tdc*, and *Str* expression in Zct1hp-38. 17β-estradiol (5μM) was added for 24 h, then 250μM or 1000μM MJ was added for the time specified (8, 24, and 48 h). Error bars represent standard deviations of qPCR triplicates.

Similar to TIA metabolite production, levels of *G10h*, *Tdc*, and *Str* increased upon treatment with 250μM MJ, but did not increase further upon treatment with 17β-estradiol and 250μM MJ (Fig 6). This trend is similarly observed in the Zct1hp-40 (S6 Fig) as well as the GFPhp-29 control line (S7 Fig). *Zct1* silencing did not affect MJ-elicited TIA gene expression. These results explain the lack of increase seen in TIA metabolite levels upon *Zct1* silencing (Fig 4).

As previously observed [16], expression levels of *G10h*, *Tdc*, and *Str* were inhibited in Zct1hp roots upon treatment with 1000μM MJ (Fig 6), and did not increase upon treatment with 17β-estradiol and 1000μM MJ. While the lack of increase is surprising, TIA production is strongly correlated with the expression of early TIA biosynthetic genes. Low TIA levels (Fig 5) are likely attributed to the low expression levels of these TIA biosynthetic genes (Fig 6).

TIA gene expression was not increased upon *Zct1* silencing of MJ-induced hairy roots either at low or high MJ dosages. Silencing *Zct1* alone was not sufficient to increase the expression of genes that ZCT1 is known to repress (i.e. *Tdc* and *Str*), suggesting that other transcription factors induced by MJ or other unidentified mechanisms contribute to the repression of TIAs.

### *Zct3* levels remained elevated with MJ elicitation

Although *Zct1* was successfully silenced, expression of TIA genes regulated by *Zct1* was unchanged (Fig 6). To investigate if other transcription factors might compensate for the effect of *Zct1* silencing, we monitored the expression of other transcription factors in *C*. *roseus*. 17β-estradiol (5μM) was added to Zct1hp-38 for 24 h followed by the addition of 250μM or 1000μM MJ for 8, 24, and 48 h. We monitored *Orca2* and *Orca3*, transcriptional activators of *Tdc* and *Str*, and *Zct2* and *Zct3*, other transcriptional repressors of *Tdc* and *Str*, through qPCR.

**Fig 7:**
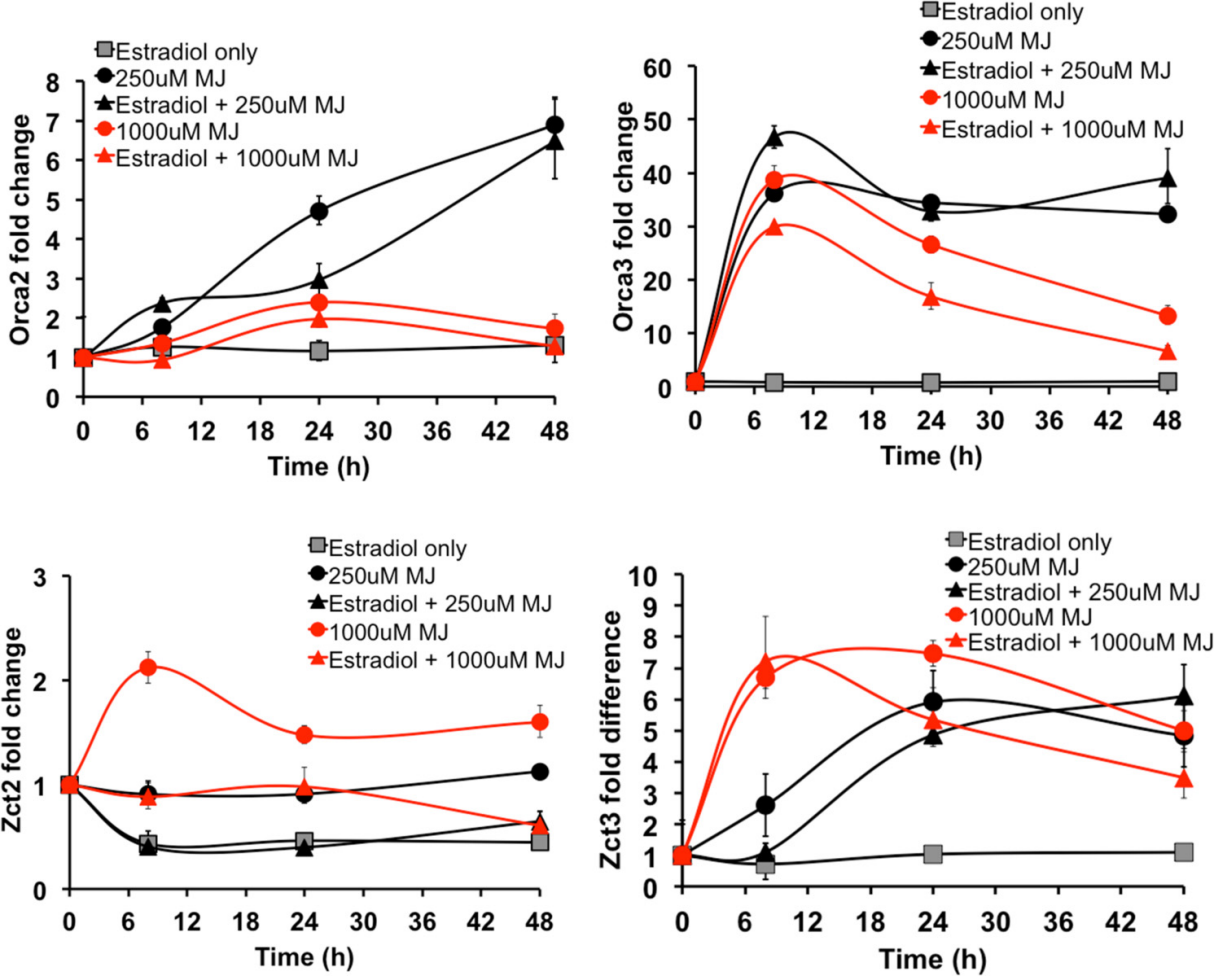
*Orca2*, *Orca3*, *Zct2*, and *Zct3* expression in Zct1hp-38. 17β-estradiol (5μM) was added for 24 h, then 250μM or 1000μM MJ was added for the time specified (8, 24, and 48 h). Error bars represent standard deviations of qPCR triplicates.

In Zct1hp roots, 250μM MJ induced *Orca2* and *Orca3* by 7 and 33-fold after 48 h, respectively, while 1000μM MJ lowered *Orca2* and *Orca3* to 2 and 12-fold (Fig 7 and S8 Fig). *Zct1* silencing did not increase *Orca* expression at the inhibitory MJ dosage, suggesting that another transcription factor may be repressing *Orca* expression at the high MJ dosage. As shown in GFPhp-29 control roots, the addition of 17β-estradiol alone did not affect *Orca* expression (S9 Fig).

We also analyzed the expression of *Zct2* and *Zct3* in the Zct1hp lines. Due to high homology, *Zct2* expression was also silenced in the Zct1hp-38 line (Fig 7) and in the Zct1hp-40 line (S8 Fig). In contrast, MJ (particularly at 1000μM) rapidly induced *Zct3* levels in Zct1hp-38, which remained high even in combination with 17β-estradiol. In the GFPhp control line, 17β-estradiol alone induced *Zct3*, suggesting that induction of RNAi itself may activate *Zct3* (S9 Fig). However, the induction by RNAi (~4-fold) is lower than with MJ-treatment (11-15-fold).

In ORCA2- and ORCA3-overexpressing hairy roots, *Zct1*, *Zct2*, and *Zct3* levels also increased [13,29], possibly counteracting the effect of the elevated *Orca* activators. In this study, *Zct1* (and *Zct2*) levels remained low through silencing, but *Zct3* levels remained elevated. Increases in TIA production may still have been limited due to high basal levels of ZCT3 (and ZCT2). Basal levels of *Zct* (particularly *Zct2* and *Zct3*, ΔCt = ~0) were more abundant than basal levels of *Orca* (particularly *Orca3*, ΔCt = -5) in the hairy root cultures (S10 Fig). Therefore, a large fold increase in *Orca3* upon MJ elicitation may not necessarily mean higher absolute transcript or protein levels than *Zct2* or *Zct3*.

Although the three ZCT proteins are thought to be functionally similar in repressing *Tdc* and *Str*, they are structurally different. For example, ZCT1 and ZCT2 are both smaller (~20 kDa) than ZCT3 (27 kDa), which also has a longer spacer between its zinc finger motifs [15]. Recently, ZCT1 and ZCT2, but not ZCT3, were shown to repress the activity of hydroxymethylbutenyl 4-diphosphate synthase (HDS) in the monoterpenoid pathway [35]. These factors suggest distinct functions of each ZCT transcriptional repressor. Since not all three ZCT repressors were *completely* silenced, the non-silenced ZCT proteins may still be limiting TIA gene expression in the absence of ZCT1. Also, since the ZCT repressors have distinct functions, silencing all three ZCTs simultaneously may be necessary to relieve their repression on alkaloid biosynthesis.

In summary, we successfully developed transgenic *C*. *roseus* hairy roots with inducible *Zct1* silencing through RNAi. Upon induction with 17β-estradiol, *Zct1* induction by MJ at both low (250μM) and high (1000μM) concentrations was considerably reduced. Despite these low levels of *Zct1*, MJ-elicited TIA production and TIA gene expression (*G10h*, *Tdc*, and *Str*) did not further increase in *Zct1* silenced cultures. Due to high homology, levels of *Zct2* (but not *Zct3*) were also silenced in *Zct1* silenced lines under MJ-elicited conditions. The ZCT repressors are thought to play overlapping but distinct functions in *C*. *roseus*. Since basal levels of *Zct2* and *Zct3* were high, silencing the expression of all three ZCTs simultaneously may be necessary to eliminate the repression by the ZCTs and to therefore increase TIA production.

## Supporting Information

S1 Fig pER8-Zct1hp plasmid map. (TIFF)

S2 Fig Genomic integration verified in transgenic *C*. *roseus* hairy roots. *Rps9* (housekeeping gene), *LexA*, *hygR*, *virD2* (*Agrobacterium* control), and *rolC* (hairy root control) genomic DNA was amplified in 10 Zct1hp and 9 GFPhp transgenic lines by PCR. WT = wild-type hairy roots, R1000 = *A*. *rhizogenes* containing pER8-Zct1hp or pER8-GFPhp plasmid, NT = no template control.
(TIFF)

S3 Fig Separation of terpenoid indole alkaloids by reverse phase HPLC. HPLC traces at 329 and 254 nm, and UV spectra of the associated peaks.
(TIFF)

S4 Fig *Zct1* expression in Zct1hp-40 and GFPhp-29 hairy roots over 24 or 48 h. 17β-estradiol (5μM) was added for 24 h, then 250μM MJ was added for the time specified. Error bars represent standard deviations of qPCR triplicates.
(TIFF)

S5 Fig TIA metabolite levels in Zct1hp-40 hairy roots. 17β-estradiol (5μM) was added for 24 h, then 250μM or 1000μM MJ was added for the time specified (3 and 7 d). The TIA were separated by HPLC and quantified by UV absorbance. Error bars represent standard deviations between two biological replicates.
(TIFF)

S6 Fig *G10h*, *Tdc*, and *Str* expression in Zct1hp-40 hairy roots. 17β-estradiol (5μM) was added for 24 h, then 250μM MJ was added for the time specified (8 and 24 h). Error bars represent standard deviations of qPCR triplicates.
(TIFF)

S7 Fig *G10h*, *Tdc*, and *Str* expression in GFPhp-29 hairy roots. 17β-estradiol (5μM) was added for 24 h, then 250μM MJ was added for the time specified (8 and 48 h). Error bars represent standard deviations of qPCR triplicates.
(TIFF)

S8 Fig *Orca2*, *Orca3*, *Zct2*, and *Zct3* expression in Zct1hp-40 hairy roots. 17β-estradiol (5μM) was added for 24 h, then 250μM MJ was added for the time specified (8 and 24 h). Error bars represent standard deviations of qPCR triplicates.
(TIFF)

S9 Fig *Orca2*, *Orca3*, *Zct2*, and *Zct3* expression in GFPhp-29 hairy roots. 17β-estradiol (5μM) was added for 24 h, then 250μM MJ was added for the time specified (8 and 48 h). Error bars represent standard deviations of qPCR triplicates.
(TIFF)

S10 Fig Basal levels of *Zct1*, *Zct2*, *Zct3*, *Orca2*, *Orca3*, *G10h*, *Tdc*, and *Str* in untreated WT and Zct1hp hairy roots. Negative values of ΔCt mean that the transcript level of *Rps9* (housekeeping gene) is higher than the transcript level of the specific gene monitored. ΔCt of 1 represents a 2-fold difference in transcript levels. Error bars represent standard deviation of three transgenic lines. Statistical significance calculated used Student’s t-test; * denotes *p*< 0.05.
(TIFF)

S1 Table Genetic engineering efforts to overexpress single or multiple TIA biosynthetic enzymes in *C*. *roseus* hairy roots. ASα = anthranilate synthase α subunit; ASβ = anthranilate synthase β subunit; TDC = tryptophan decarboxylase; DXS = 1-deoxy-D-xylulose-synthase; G10H = geraniol-10-hydroxylase; DAT = deacetylvindoline 4-O-acetyltransferase.
(DOCX)

S2 Table Primers used for cloning *Zct1* hairpin into pER8 and antibiotic resistance conferred by each plasmid. (DOCX)

S3 Table Primer sequences used to check genomic integration. *primer sequences were adapted from [6]. **primer sequences were adapted from [7].
(DOCX)

S4 Table Primer sequences used for qPCR analysis of *C*. *roseus* transcription factor and TIA biosynthetic genes. *Orca* and *Zct* primers were previously described in [8]. *Tdc* and *G10h* primers were previously described in [9]. *Str* primers are newly designed.
(DOCX)
