# Supplementary figures and images for: Silencing the Transcriptional Repressor, ZCT1, Illustrates the Tight Regulation of Terpenoid Indole Alkaloid Biosynthesis in Catharanthus roseus Hairy Roots
Source: PLoS One. 2016 Jul 28;11(7):e0159712. doi: 10.1371/journal.pone.0159712 (PMC4965073; doi:10.1371/journal.pone.0159712)

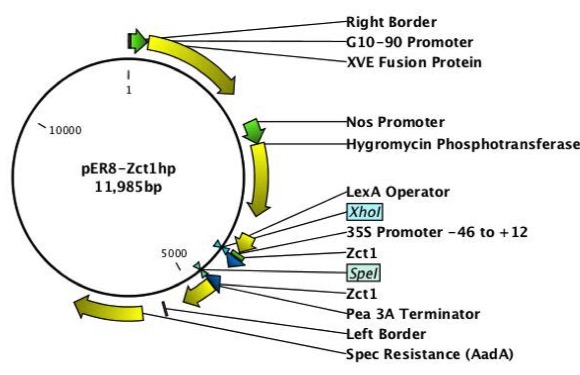

Supplement: S1 Fig — (TIFF) [file pone.0159712.s001.tiff]

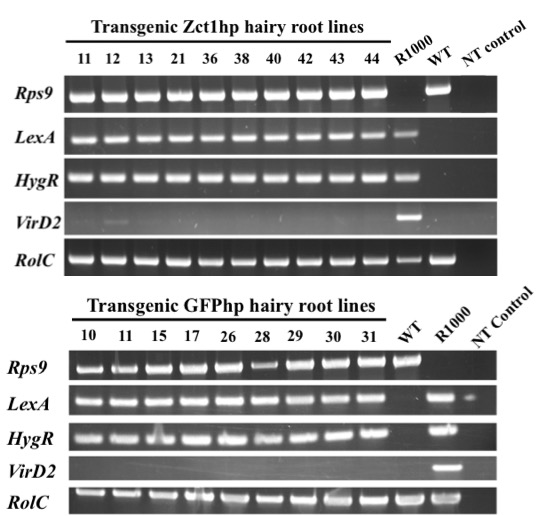

Supplement: S2 Fig — Rps9 (housekeeping gene), LexA, hygR, virD2 (Agrobacterium control), and rolC (hairy root control) genomic DNA was amplified in 10 Zct1hp and 9 GFPhp transgenic lines by PCR. WT = wild-type hairy roots, R1000 = A. rhizogenes containing pER8-Zct1hp or pER8-GFPhp plasmid, NT = no template control. (TIFF) [file pone.0159712.s002.tiff]

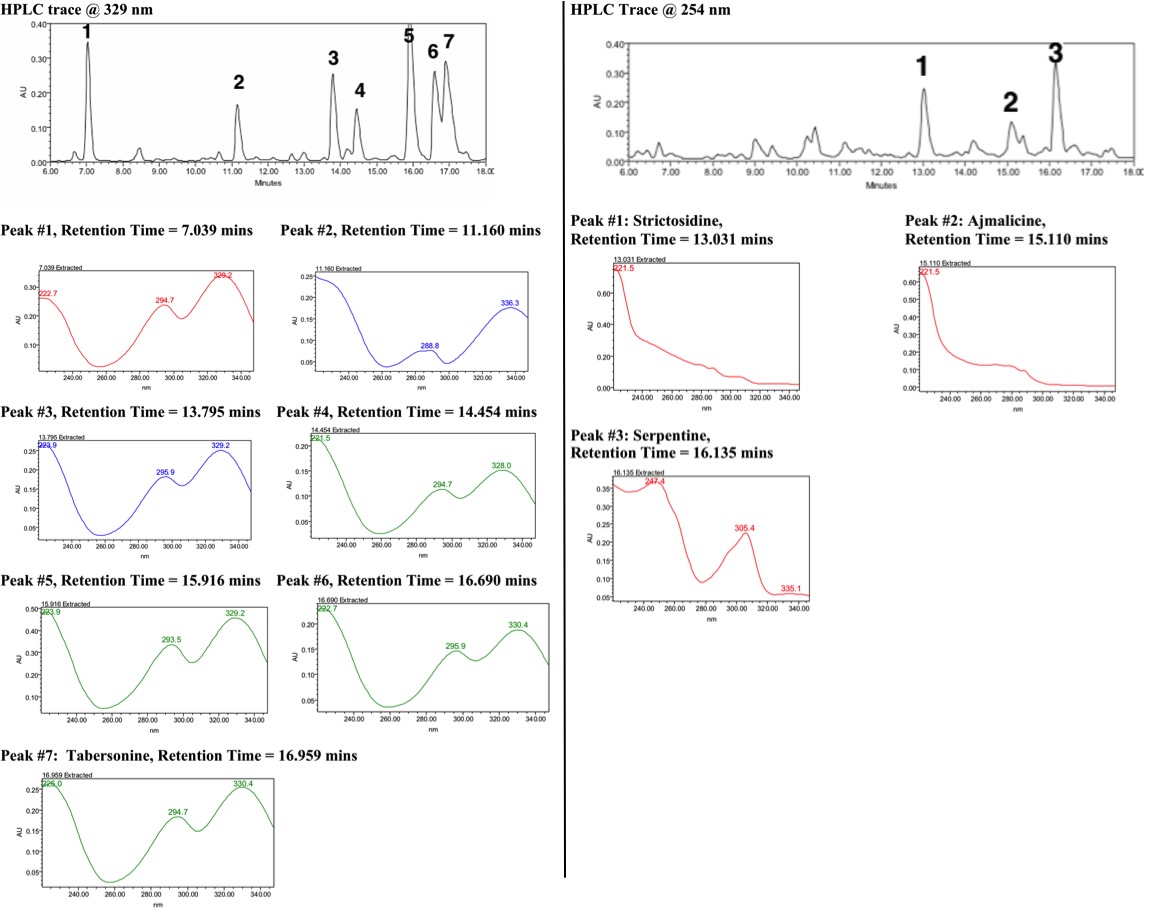

Supplement: S3 Fig — HPLC traces at 329 and 254 nm, and UV spectra of the associated peaks. (TIFF) [file pone.0159712.s003.tiff]

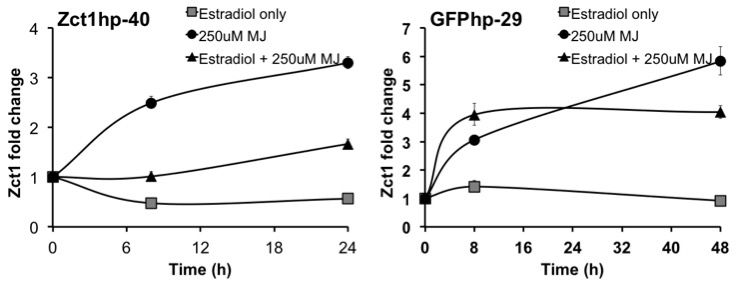

Supplement: S4 Fig — 17β-estradiol (5μM) was added for 24 h, then 250μM MJ was added for the time specified. Error bars represent standard deviations of qPCR triplicates. (TIFF) [file pone.0159712.s004.tiff]

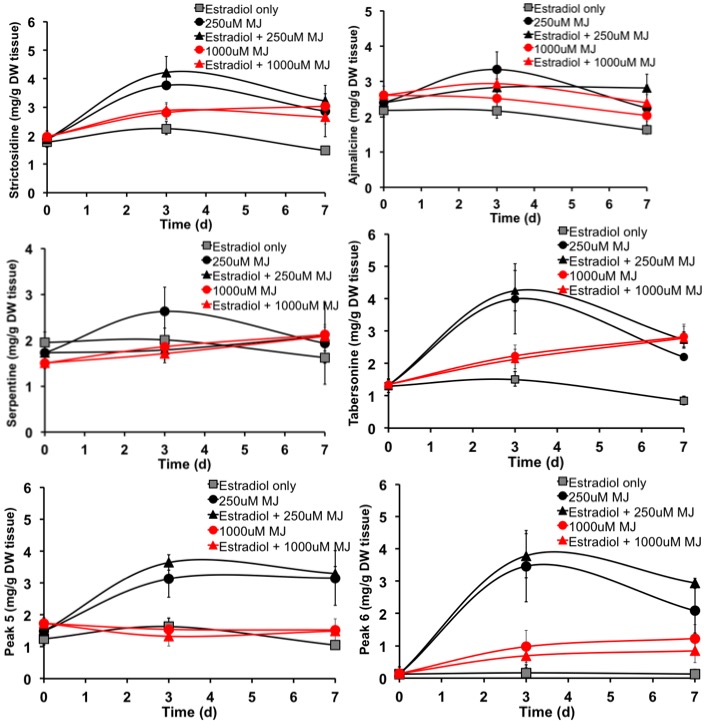

Supplement: S5 Fig — 17β-estradiol (5μM) was added for 24 h, then 250μM or 1000μM MJ was added for the time specified (3 and 7 d). The TIA were separated by HPLC and quantified by UV absorbance. Error bars represent standard deviations between two biological replicates. (TIFF) [file pone.0159712.s005.tiff]

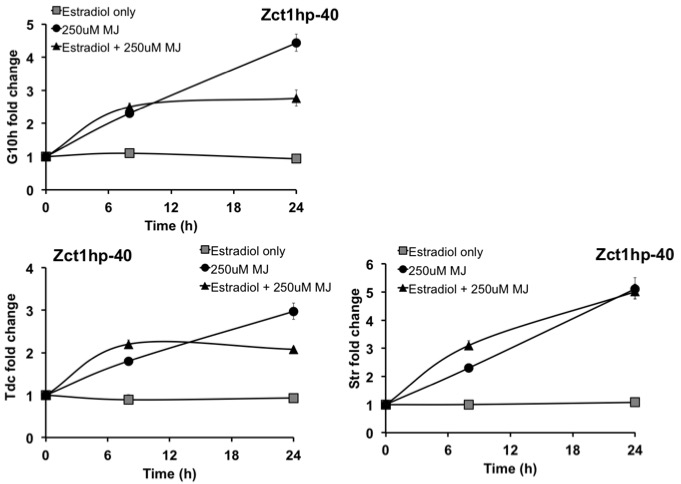

Supplement: S6 Fig — 17β-estradiol (5μM) was added for 24 h, then 250μM MJ was added for the time specified (8 and 24 h). Error bars represent standard deviations of qPCR triplicates. (TIFF) [file pone.0159712.s006.tiff]

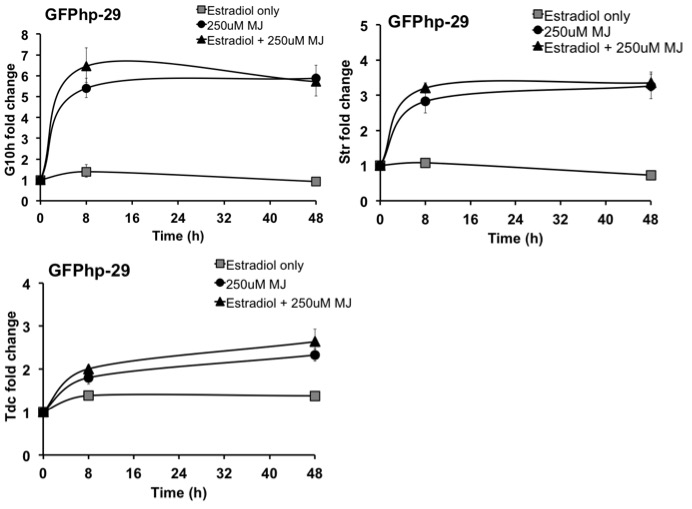

Supplement: S7 Fig — 17β-estradiol (5μM) was added for 24 h, then 250μM MJ was added for the time specified (8 and 48 h). Error bars represent standard deviations of qPCR triplicates. (TIFF) [file pone.0159712.s007.tiff]

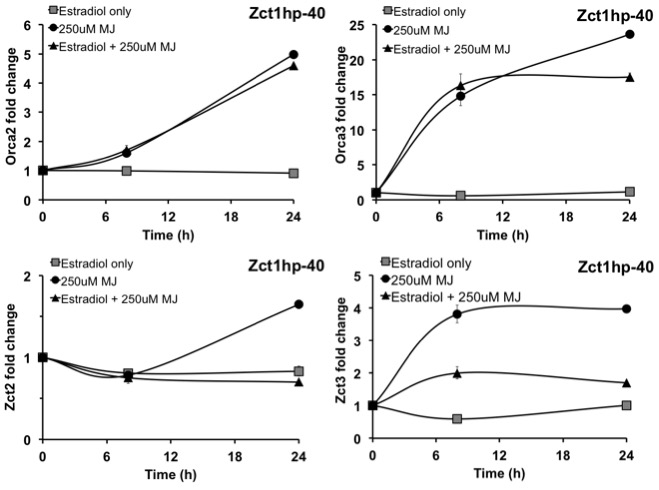

Supplement: S8 Fig — 17β-estradiol (5μM) was added for 24 h, then 250μM MJ was added for the time specified (8 and 24 h). Error bars represent standard deviations of qPCR triplicates. (TIFF) [file pone.0159712.s008.tiff]

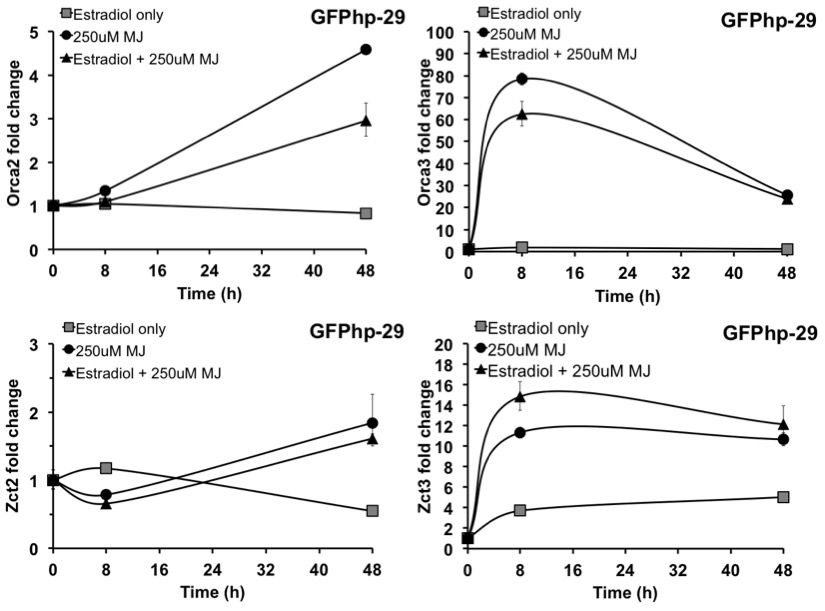

Supplement: S9 Fig — 17β-estradiol (5μM) was added for 24 h, then 250μM MJ was added for the time specified (8 and 48 h). Error bars represent standard deviations of qPCR triplicates. (TIFF) [file pone.0159712.s009.tiff]

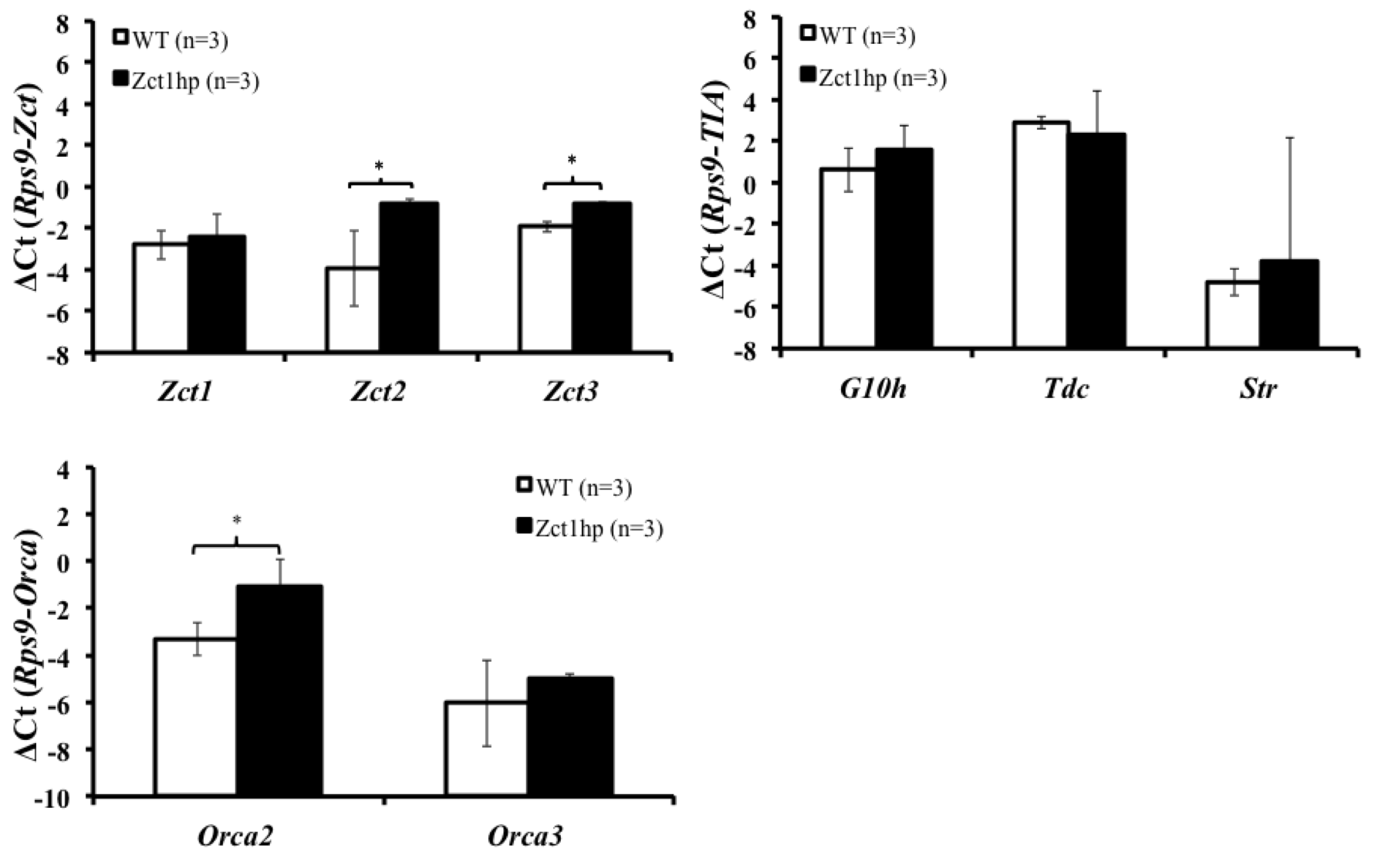

Supplement: S10 Fig — Negative values of ΔCt mean that the transcript level of Rps9 (housekeeping gene) is higher than the transcript level of the specific gene monitored. ΔCt of 1 represents a 2-fold difference in transcript levels. Error bars represent standard deviation of three transgenic lines. Statistical significance calculated used Student’s t-test; * denotes p< 0.05. (TIFF) [file pone.0159712.s010.tiff]
