# Supplementary material for: Silencing the Transcriptional Repressor, ZCT1, Illustrates the Tight Regulation of Terpenoid Indole Alkaloid Biosynthesis in Catharanthus roseus Hairy Roots
Source: PLoS One. 2016 Jul 28;11(7):e0159712. doi: 10.1371/journal.pone.0159712 (PMC4965073; doi:10.1371/journal.pone.0159712)
Supplement: S1 Table — ASα = anthranilate synthase α subunit; ASβ = anthranilate synthase β subunit; TDC = tryptophan decarboxylase; DXS = 1-deoxy-D-xylulose-synthase; G10H = geraniol-10-hydroxylase; DAT = deacetylvindoline 4-O-acetyltransferase. (DOCX) [file pone.0159712.s011.docx]

| **Pathway** | **TIA biosynthetic gene(s) overexpressed** | **Outcome** | **Reference** |
| --- | --- | --- | --- |
| Indole pathway | Feedback-insensitive *Asα* | * 300-fold increase in tryptophan * 10-fold increase in tryptamine * 1.7-fold increase of lochnericine | [1] |
|  | *Tdc* | * no significant increase in tryptamine * 1.3-fold increase in serpentine | [2] |
|  | Feedback-insensitive *Asα* + *Tdc* | * 6-fold increase in tryptamine * no increase in downstream TIAs |  |
|  | Feedback-insensitive *Asα* + *Asβ* | * 40-fold increase in tryptophan * 8-fold increase in tryptamine * varied results on TIA levels | [3] |
|  | Feedback-insensitive *Asα + Asβ* + *Tdc* | * 14-fold increase in tryptophan * 3-fold increase in trypamine |  |
| Terpenoid pathway | *Dxs* | * 1.7-fold increase in levels of ajmalicine and lochnericine * decreased levels of tabersonine and horhammericine | [4] |
|  | *Dxs* and *G10h* | * 1.4-fold increase in tabersonine * 1.2-fold increase in lochnericine * overall positive gain in metabolites compared to the mixed results of overexpressing DXS alone |  |
| Terpenoid  and indole pathways | *Dxs* and feedback-resistant *Asα* | * 1.2-fold increase in tabersonine * 1.2-fold increase in lochnericine |  |
| Downstream | *Dat* | * 4-fold increase in levels of horhammericine * no change in levels of other TIAs | [5] |

S1 Table: Genetic engineering efforts to overexpress single or multiple TIA biosynthetic enzymes in *C. roseus* hairy roots. ASα = anthranilate synthase α subunit; ASβ = anthranilate synthase β subunit; TDC = tryptophan decarboxylase; DXS = 1-deoxy-D-xylulose-synthase; G10H = geraniol-10-hydroxylase; DAT = deacetylvindoline 4-O-acetyltransferase

1. Hughes EH, Hong SB, Gibson SI, Shanks JV, San KY (2004a) Expression of a feedback-resistant anthranilate synthase in *Catharanthus roseus* hairy roots provides evidence for tight regulation of terpenoid indole alkaloid levels. Biotechnol Bioeng 86: 718-727.
2. Hughes EH, Hong SB, Gibson SI, Shanks JV, San KY (2004b) Metabolic engineering of the indole pathway in *Catharanthus roseus* hairy roots and increased accumulation of tryptamine and serpentine. Metab Eng 6: 268-276.
3. Hong SB, Peebles CA, Shanks JV, San KY, Gibson SI (2006) Expression of the Arabidopsis feedback-insensitive anthranilate synthase holoenzyme and tryptophan decarboxylase genes in *Catharanthus roseus* hairy roots. J Biotechnol 122: 28-38.
4. Peebles CA, Sander GW, Hughes EH, Peacock R, Shanks JV, et al. (2011) The expression of 1-deoxy-d-xylulose synthase and geraniol-10-hydroxylase or anthranilate synthase increases terpenoid indole alkaloid accumulation in *Catharanthus roseus* hairy roots. Metabolic engineering 13: 234-240.
5. Magnotta M, Murata J, Chen J, De Luca V (2007) Expression of deacetylvindoline-4-O-acetyltransferase in *Catharanthus roseus* hairy roots. Phytochemistry 68: 1922-1931.
