# Supplementary material for: Silencing the Transcriptional Repressor, ZCT1, Illustrates the Tight Regulation of Terpenoid Indole Alkaloid Biosynthesis in Catharanthus roseus Hairy Roots
Source: PLoS One. 2016 Jul 28;11(7):e0159712. doi: 10.1371/journal.pone.0159712 (PMC4965073; doi:10.1371/journal.pone.0159712)
Supplement: S2 Table — (DOCX) [file pone.0159712.s012.docx]

| **Primers for cloning *Zct1* in MCS1 of pSK-Int** | |
| --- | --- |
| **Primer** | **Sequence (5' to 3')** |
| *Zct1F*XhoI | TAACTCGAGGGCGGCAGATCTACTCCATCAATC |
| *Zct1R*HindIII | TAAAAGCTTATCACTCACCACCACCGGTTTCTC |

| **Primers for cloning *Zct1* into MCS2 of pSK-Int** | |
| --- | --- |
| **Primer** | **Sequence (5’ to 3’)** |
| *Zct1F*SpeI | TAAACTAGTGGCGGCAGATCTACTCCATCAATC |
| *Zct1R*EcoRI | TAAGAATTCATCACTCACCACCACCGGTTTCTC |

| **Vector Name** | **Antibiotic Resistance** | **Concentration used** |
| --- | --- | --- |
| pSK-Int | Ampicillin | 40 mg/L |
| pER8 | Spectinomycin | 100 mg/L |
| pUC57(kan) | Kanamycin | 40 mg/L |

S2 Table: Primers used for cloning *Zct1* hairpin into pER8 and antibiotic resistance conferred by each plasmid.
