# Supplementary material for: Silencing the Transcriptional Repressor, ZCT1, Illustrates the Tight Regulation of Terpenoid Indole Alkaloid Biosynthesis in Catharanthus roseus Hairy Roots
Source: PLoS One. 2016 Jul 28;11(7):e0159712. doi: 10.1371/journal.pone.0159712 (PMC4965073; doi:10.1371/journal.pone.0159712)
Supplement: S3 Table — *primer sequences were adapted from [6]. **primer sequences were adapted from [7]. (DOCX) [file pone.0159712.s013.docx]

| **Primer** | **Sequence (5’ to 3’)** |
| --- | --- |
| *Rps9_F* | TCCACCATGCCAGAGTGCTCATTA |
| *Rps9_R* | TCCATCACCACCAGATGCCTTCTT |
| *LexA_F* | ATCCCCTCGACGTACTGTAC |
| *LexA_R* | TTGCGAAGATCCTAGAGTCGA |
| *hygR_F187* | GTTTATCGGCACTTTGCATC |
| *hygR_R447* | TCACGCCATGTAGTGTATTG |
| *virD2_F1** | ATGCCCGATCGAGCTCAAGT |
| *virD2_R388** | CCTGACCCAAACATCTCGGCTGCCCA |
| *RolC_F*** | CAACCTGTTTCCTACTTTGTTAAC |
| *RolC_R*** | AAACAAGTGACACACTCAGCTTC |

S3 Table: Primer sequences used to check genomic integration. *primer sequences were adapted from [6]. **primer sequences were adapted from [7].

6. Haas JH, Moore LW, Ream W, Manulis S (1995) Universal PCR primers for detection of phytopathogenic *Agrobacterium* strains. Applied and Environmental Microbiology 61: 2879-2884.

7. Suttipanta N, Pattanaik S, Kulshrestha M, Patra B, Singh SK, et al. (2011) The transcription factor CrWRKY1 positively regulates the terpenoid indole alkaloid biosynthesis in *Catharanthus roseus*. Plant Physiology 157: 2081-2093.
