# Supplementary material for: Silencing the Transcriptional Repressor, ZCT1, Illustrates the Tight Regulation of Terpenoid Indole Alkaloid Biosynthesis in Catharanthus roseus Hairy Roots
Source: PLoS One. 2016 Jul 28;11(7):e0159712. doi: 10.1371/journal.pone.0159712 (PMC4965073; doi:10.1371/journal.pone.0159712)
Supplement: S4 Table — Orca and Zct primers were previously described in [8]. Tdc and G10h primers were previously described in [9]. Str primers are newly designed. (DOCX) [file pone.0159712.s014.docx]

| **Primer** | **Sequence (5’ to 3’)** |
| --- | --- |
| *Orca2_F* | GAAATTCGCTGCGGAAATCAGGGA |
| *Orca2_R* | AGATGACACGATGAAGATCGGCGT |
| *Orca3_F* | TGTCAGGAGGATTCTGTTGTGGGA |
| *Orca3_R* | CGCATATTAAACGCGGCTGCATCA |
| *Zct1_F* | AATCTTTAGCGGTGACGAAGCCGA |
| *Zct1_R* | CGTTGTCCTCAGGCGTCAAATTCA |
| *Zct2_F* | TTTCCATCGTTTCAAGCTCTCGGC |
| *Zct2_R* | ATTACCGGACGCCGAATCACTCAT |
| *Zct3_F* | CAGCAACAACAACCCACCGAAGAA |
| *Zct3_R* | TTGCCTTATGTCCTCCGAGTGCTT |
| *Tdc_F* | ACCTACGACCGTCGAAACGGATTT |
| *Tdc_R* | AAACTCGGGACATATACAGGCGCT |
| *Str_F* | GCTAGAAGGGCCAAAGAA |
| *Str_R* | GGTGGTGGAAGTGGTATAA |
| *G10h_F* | TAGCAGGGACGGACACAACATCAA |
| *G10h_R* | TCACGTCCAATTGCCCAAGCATTC |

S4 Table: Primer sequences used for qPCR analysis of *C. roseus* transcription factor and TIA biosynthetic genes. *Orca* and *Zct* primers were previously described in [8]. *Tdc* and *G10h* primers were previously described in [9]. *Str* primers are newly designed.

8. Goklany S, Rizvi NF, Loring RH, Cram EJ, Lee-Parsons CWT (2013) Jasmonate-dependent alkaloid biosynthesis in *Catharanthus roseus* hairy root cultures is correlated with the relative expression of *Orca* and *Zct* transcription factors. Biotechnology Progress 29: 1367-1376.

9. Goklany S, Loring RH, Glick J, Lee-Parsons CWT (2009) Assessing the limitations to terpenoid indole alkaloid biosynthesis in *Catharanthus roseus* hairy root cultures through gene expression profiling and precursor feeding. Biotechnology Progress 25: 1289 - 1296.
